# Cross-cultural adaptation of the VISA-A questionnaire, an index of clinical severity for patients with Achilles tendinopathy, with reliability, validity and structure evaluations

**DOI:** 10.1186/1471-2474-6-12

**Published:** 2005-03-06

**Authors:** Karin Grävare Silbernagel, Roland Thomeé, Jon Karlsson

**Affiliations:** 1 Lundberg Laboratory of Orthopaedic Research, Dept of Orthopaedics, Göteborg University, Sahlgrenska University Hospital, Göteborg, Sweden; 2 Sportrehab – Physical Therapy & Sports Medicine Clinic, Göteborg, Sweden; 3 Dept of Orthopaedics, Sahlgrenska University Hospital, Göteborg, Sweden

## Abstract

**Background:**

Achilles tendinopathy is considered to be one of the most common overuse injuries in elite and recreational athletes and the recommended treatment varies. One factor that has been stressed in the literature is the lack of standardized outcome measures that can be used in all countries. One such standardized outcome measure is the Victorian Institute of Sports Assessment – Achilles (VISA-A) questionnaire, which is designed to evaluate the clinical severity for patients with Achilles tendinopathy. The purpose of this study was to cross-culturally adapt the VISA-A questionnaire to Swedish, and to perform reliability, validity and structure evaluations.

**Methods:**

Cross-cultural adaptation was performed in several steps including translations, synthesis of translations, back translations, expert committee review and pre-testing. The final Swedish version, the VISA-A Swedish version (VISA-A-S) was tested for reliability on healthy individuals (n = 15), and patients (n = 22). Tests for internal consistency, validity and structure were performed on 51 patients.

**Results:**

The VISA-A-S had good reliability for patients (*r *= 0.89, ICC = 0.89) and healthy individuals (*r *= 0.89–0.99, ICC = 0.88–0.99). The internal consistency was 0.77 (Cronbach's alpha). The mean [95% confidence interval] VISA-A-S score in the 51 patients (50 [44–56]) was significantly lower than in the healthy individuals (96 [94–99]). The VISA-A-S score correlated significantly (Spearman's *r *= -0.68) with another tendon grading system. Criterion validity was considered good when comparing the scores of the Swedish version with the English version in both healthy individuals and patients. The factor analysis gave the factors *pain/symptoms *and *physical activity*

**Conclusion:**

The VISA-A-S questionnaire is a reliable and valid instrument and comparable to the original version. It measures two factors: *pain/symptoms *and *physical activity*, and can be used in both research and the clinical setting.

## Background

Achilles tendinopathy is a common overuse injury, especially among athletes involved in activities that include running and jumping [1-4]. Several studies report the incidence of Achilles tendon disorders in runners to be 6–18% of all injuries [3,5,6]. Most commonly afflicted are middle-aged men, but Achilles tendinopathy occurs in both men and women at various ages [2,4,7-9]. Common complaints are pain during and after physical activity, tenderness on palpation and morning stiffness [7-10]. Symptoms usually subside with decreased physical activity, but tend to return as soon as physical activity is increased [2]. With increased severity patients may also have pain during daily functional activities [7,10]. Achilles tendinopathy causes many patients to significantly decrease their physical activity level, with a potentially negative impact on their overall health and general well-being [2,3,7].

Despite the high incidence of Achilles tendon disorders there have been few randomized treatment studies in patients with Achilles tendinopathy [8,11-16]. It is also difficult to compare the results of studies as outcome measures vary widely. A universally used clinical outcome measure of the symptoms and function would help comparisons between treatments in various clinics and research studies, and could also be the basis of criteria for various treatments.

Robinson et al. [17] developed a questionnaire as an index of clinical severity of Achilles tendinopathy; the Victorian Institute of Sports Assessment – Achilles questionnaire (VISA-A). The VISA-A questionnaire is an easily self-administered questionnaire that evaluates symptoms and their effect on physical activity. It can be used to compare different populations with Achilles tendinopathy, and facilitate comparisons between studies. The VISA-A score has already been used to evaluate the outcome of treatment [16]. In the clinic, the VISA-A questionnaire can be used to determine the patient's clinical severity and provide a guideline for treatment as well as for monitoring the effect of treatment. In order to use the VISA-A questionnaire for non-english speaking patients it needs to be translated, culturally adapted and properly evaluated [18].

Therefore, the purpose of this study was to translate and culturally adapt the English VISA-A questionnaire to Swedish, to perform reliability and validity evaluations of the Swedish version, and to analyze the factor structure of the questionnaire.

## Methods

To establish good face validity and content validity, the translation and cultural adaptation of the VISA-A questionnaire into Swedish was performed in several steps [18]. The English version was translated into Swedish independently by three people. All three were working in the medical field and had English as a second language. The three translations were synthesized into one Swedish version by a panel of experts consisting of four physical therapists who specialize in musculoskeletal disorders. The back translations of the Swedish version into English were performed by another three people. Two of the back translators were in the medical field (a sports medicine doctor and a physical therapist), and the third person was Swedish but has lived in the USA for many years. The panel of experts (same as above) then compared the original version with the back translations.

The panel of experts consolidated the various versions into one pre-final version of the VISA-A questionnaire – Swedish version (VISA-A-S). The pre-VISA-A-S was pilot tested on five patients and five healthy individuals. After pilot testing question 1 was made clearer by entering the minutes in the boxes.

The final version of the VISA-A-S (Additional file 1) was tested on both healthy individuals and patients with a diagnosis of Achilles tendinopathy. All subjects were given written information about the purpose and procedure of the study, and informed consent was obtained. Ethics approval was obtained from the Ethics Committee at the Medical Faculty, Gothenburg University, Sweden.

For test-retest evaluation, we recruited a convenience sample of 15 healthy individuals (Table 1), age 20–40 years. They completed the VISA-A-S questionnaire three times within two weeks. The questions were answered with respect to their right Achilles tendon.

**Table 1:** Summary of study populations Mean, standard deviation (SD) and 95 % confidence interval (CI) for age, duration of symptoms and VISA-A-S score for the study populations.

	Age (years)			Duration of symptoms (months)			VISA-A-S Score		
	
	Mean	SD	95% CI	Mean	SD	95% CI	Mean	SD	95% CI
Healthy (n = 15, 3 F, 12 M)	29.5	4.3	27.1 – 31.9	N/A	N/A	N/A	96	4	94 – 99
**Reliability Group** Patients (n = 22, 8 F, 14 M)	45.4	15.5	38.6 – 52.3	55.5	134.7	6.3 – 57.4	50	24	40 – 61
**Validity Group** Patients (n = 51, 19 F, 32 M)	43.1	14.5	39.0 – 47.2	31.8	90.8	6.3 – 57.4	50	23	44 – 56

(N/A = not applicable, F = females, M = men)

Fifty-one patients (Table 1), 39 to 47 years old, with Achilles tendinopathy, were included in the reliability evaluation for internal consistency and the validity evaluations. Twenty-two (Table 1) of the 51 patients also participated in a test-retest evaluation. Bilateral symptoms were reported in 15 of 51 patients (7 of the 22 in test-retest group). The patients were recruited from 11 physical therapy clinics throughout Sweden. The inclusion criteria were the same as in the original study [17]. The subjects had to be older than 18 and be able to give written consent. The subjects had to have a diagnosis of Achilles tendinosis, paratendinitis, or partial rupture with or without a retrocalcaneal or Achilles bursitis. The diagnosis was based on patient history and the physical therapists' clinical findings. Subjects with total Achilles tendon rupture and pregnant or nursing women were excluded. At their first physical therapy visit, all patients completed questionnaires regarding their injury, physical activity [19], tendon injury according to Stanish [1], and one VISA-A-S questionnaire for each leg. For the patients with bilateral symptoms, the side with the lowest VISA-A-S score, or if the score was equal, the side with the longest duration of symptoms was chosen for the evaluations. The 22 patients that participated in the test-retest evaluation completed the VISA-A-S questionnaire a second time within a week of the first visit.

Construct validity of the VISA-A-S was tested according to the original article on the VISA-A English version [17]. The results from the 51 patients who completed the VISA-A-S questionnaire were compared with the results from the tendon grading system by Stanish et al. (1984). The results from the VISA-A-S questionnaire for patients with Achilles tendinopathy were also compared with the results of healthy individuals.

Criterion validity of the VISA-A-S questionnaire was evaluated by comparing the results of our patients (n = 51) with the results of the two patient groups, the non-surgical group (n = 45) and surgical group (n = 14), in the original article by Robinson et al. (2001). The results of the healthy individuals in our study were also compared with the results from the healthy individuals in the original study.

The structure of the VISA-A-S questionnaire was evaluated with a factor analysis.

### Statistical analysis

All data were analysed by SPSS 11.5 for Windows. Descriptive data are reported as mean, standard deviation and 95% confidence interval.

Test-retest data was analysed by Pearson's *r*, as performed for the VISA-A English version [17]. Inter-Class Correlation Coefficient (ICC) and Wilcoxon paired test for non-parametric data was also calculated for test-retest data since the questionnaire presents ordinal data. Internal consistency was assessed by calculation of Cronbach's alpha.

Comparison of VISA-A-S with Stanish et al. (2000) tendon grading system was performed by calculating the Spearman's rank correlation coefficient for non-parametric data. VISA-A-S scores for the healthy group and the patient group were compared using the Mann Whitney *U *test. For comparison of the VISA-A-S with the VISA-A, a two sample t-test was used since only means and standard deviations, and no raw data, were available from the results in the original study [17]. The level of significance was set at p < 0.05.

A principal axis factoring with varimax rotation, eigenvalue over 1.0, was applied for evaluation of the structure of the questionnaire.

## Results

Table 2 summarizes the reliability evaluation of the VISA-A-S questionnaire. The VISA-A-S showed good test-retest reliability for healthy individuals (Pearson's *r *> 0.88, ICC > 0.88) and for patients (Pearson's *r *= 0.89, ICC = 0.89). There was no significant difference between the scores on test days 1 and 2. When analyzing each question separately (Table 3) the results showed good reliability. For questions 3 and 6, however, there were significant differences (p = 0.007 and p = 0.03 respectively) between the two test occasions. The internal consistency for the 8 questions in the VISA-A-S was 0.77 as measured with Cronbach's alpha.

**Table 2:** Summary of reliability tests of VISA-A-S score

	Pearson's *r*	ICC	Wilcoxon	Cronbach's alpha
Healthy (n = 15)				
Test-retest 1–2	0.88	0.88	0.07	
2–3	0.99	0.99	0.32	
1–3	0.90	0.90	0.07	

Patients (n = 22) Test-retest 1 week	0.89	0.89	0.051	

Patients (n = 51) Internal consistency				0.77

**Table 3:** Test-retest scores for the 8 questions in the VISA-A-S score (patients, n = 22)

	**Pearson's *r***	**ICC**	**Wilcoxon**
Question 1	0.74	0.72	0.184
Question 2	0.81	0.81	0.308
Question 3	0.89	0.86	**0.007**
Question 4	0.78	0.78	0.269
Question 5	0.71	0.71	0.793
Question 6	0.68	0.66	**0.032**
Question 7	0.87	0.86	0.577
Question 8	0.79	0.79	0.721

The VISA-A-S score correlated significantly with the tendon grading system by Stanish et al. (2000) (Spearman's *r *= -0.68; p < 0.01). The patients with Achilles tendinopathy had a significantly lower score (p < 0.0001) compared with the healthy individuals. The mean VISA-A-S score for patients in the present study was significantly (p < 0.01) lower than the mean VISA-A score for the non-surgical group in the original article by Robinson et al. (2001). When comparing the VISA-A-S score for patients in the present study with the VISA-A score of the surgical group in the original study [17] there was no significant difference (p > 0.2). There was no significant (p > 0.2) difference between the healthy individuals score on the VISA-A-S when comparing with the healthy individuals in the original article [17].

The factor analysis revealed two factors of importance (eigenvalue over 1.0): *pain/symptoms *(questions 1–6) and *physical activity *(questions 7 and 8).

## Discussion

A widely-used clinical outcome measure for patients with Achilles tendinopathy would help comparisons between treatments in various clinics and research studies. The VISA-A questionnaire is an easily self-administered questionnaire. Since research is performed in various countries it is important to properly translate, culturally adapt and evaluate instruments like a questionnaire in order to be able to compare the results [18].

This study demonstrates that the Swedish version of the VISA-A questionnaire has good reliability and validity. With careful translation and cultural adaptation, we established good *face validity *and *content validity*. The *test-retest reliability *and the *internal consistency *were considered good. A significant and strong correlation between the VISA-A-S and the tendon grading system by Stanish et al. (2000) indicates good *construct validity*. The comparison of the results of the patients and healthy individuals in the present study with the results of the non-surgical group, the surgical group and the healthy individuals, as reported in the original article by Robinson et al. (2001), indicates good *criterion validity*.

The factor analysis gave the two factors: *pain/symptoms *(questions 1–6) and *physical activity *(questions 7 and 8), strongly confirming that the questionnaire is valid for evaluating the patient's symptoms and its effect on physical activity. The factor analysis and an internal consistency of 0.77 as measured by Cronbach's alpha indicate that no question should be excluded.

We did not include a separate group of pre-surgical patients as in the original study by Robinsson et al. (2001). This is because the advances in the non-surgical rehabilitation of patients with Achilles tendinopathy during recent years have resulted in markedly reduced number of patients awaiting surgery. The patients in the present study can therefore be viewed as representing patients from both groups (surgical and non-surgical) of patients used in the original study [17]. This would explain why the patient group in the present study had a significantly lower score when compared to the non-surgical group in the original article [17].

The one week duration between the two tests in the test-retest reliability part was somewhat long. This duration was chosen because this is the usual time that lapses between a patient's first and second visit with the physical therapist. This may explain why two of the questions differed significantly between test-days. During this week the patients have met with their physical therapist, which may have caused the patients to change their view on their symptoms and physical ability.

Good criterion validity indicates that the Swedish version and the English version of the VISA-A questionnaire evaluate the same aspects of clinical severity in patients with Achilles tendinopathy. It can, thus, be expected that similar scores in the two versions indicate the same index of severity in patients with Achilles tendinopathy.

The 11 physical therapy clinics throughout Sweden, which participated in this study, all reported that the questionnaire was easily administered, and required a minimum of communication between the physical therapist and the patient. The physical therapists perceived the questionnaire as a good clinical tool and useful when treating patients with Achilles tendinopathy.

A review of the literature in regards to treatment of Achilles tendinopathy yielded only a few randomized treatment trials [8,11-16]. There are however prospective and retrospective cohort studies as well as case studies [4,20-25]. Comparing the results of all these studies is difficult since the outcome measures vary. A questionnaire like the VISA-A and VISA-A-S which gives an index of the clinical severity for patients with Achilles tendinopathy, and also is easily administered and easy to fill out, could be very helpful in the future. Paavola et al. (2002) noted that few randomized intervention studies had a follow-up longer than twelve months. The VISA-A and VISA-A-S questionnaire can be filled out easily and quickly and require a minimum of assistance during follow-up and could therefore be very helpful for long-term follow-ups. The VISA-A questionnaire has successfully been used as an outcome measure in a randomized double-blind, placebo-controlled treatment trial [16]. Currently we are evaluating the VISA-A-S questionnaires responsiveness over time in a randomized treatment study for patients with Achilles tendinopathy.

## Conclusion

This study has carefully performed the recommended steps for cross-cultural adaptation and has performed reliability and validity evaluations of the new version. The factor analysis, measuring the two factors: *pain/symptoms *and *physical activity*, reinforces that the VISA-A questionnaire can be used as an index of clinical severity. The present study culturally adapts and validates the VISA-A-S questionnaire (Additional file 1) for the Scandinavian countries and it is comparable to the original version.

## Competing interests

The author(s) declare that they have no competing interests.

## Authors' contributions

KGS conceived of the study, participated in its design, performed data acquisition, analyzed and interpreted the data and drafted the manuscript

RT conceived the study, participated in its design, interpreted the data and helped to draft the manuscript.

JK participated in the study design, interpreted the data and helped to draft the manuscript.

All authors read and approved the final manuscript.

## Pre-publication history

The pre-publication history for this paper can be accessed here:



## Supplementary Material

Additional File 1 **VISA-A-S questionnaire **The Swedish version of the VISA-A questionnaire in MICROSOFT WORD format. Click here for file

## References

[B1] Stanish WD, Curwin S, Mandell S (2000). Tendinitis: its etiology and treatment..

[B2] Kvist M (1994). Achilles tendon injuries in athletes. Sports Med.

[B3] Józsa L, Kannus P (1997). Human tendons. Anatomy, physiology and pathology..

[B4] Paavola M, Kannus P, Paakkala T, Pasanen M, Jarvinen M (2000). Long-term prognosis of patients with achilles tendinopathy. An observational 8-year follow-up study. Am J Sports Med.

[B5] James SL, Bates BT, Osternig LR (1978). Injuries to runners. Am J Sports Med.

[B6] Clement DB, Taunton JE, Smart GW (1984). Achilles tendinitis and peritendinitis: etiology and treatment. Am J Sports Med.

[B7] Paavola M, Kannus P, Jarvinen TA, Khan K, Jozsa L, Jarvinen M (2002). Achilles tendinopathy. J Bone Joint Surg Am.

[B8] Silbernagel KG, Thomeé R, Thomeé P, Karlsson J (2001). Eccentric overload training for patients with chronic Achilles tendon pain--a randomised controlled study with reliability testing of the evaluation methods. Scand J Med Sci Sports.

[B9] Alfredson H (2003). Chronic midportion Achilles tendinopathy: an update on research and treatment. Clin Sports Med.

[B10] Kader D, Saxena A, Movin T, Maffulli N (2002). Achilles tendinopathy: some aspects of basic science and clinical management. Br J Sports Med.

[B11] Mafi N, Lorentzon R, Alfredson H (2001). Superior short-term results with eccentric calf muscle training compared to concentric training in a randomized prospective multicenter study on patients with chronic Achilles tendinosis. Knee Surg Sports Traumatol Arthrosc.

[B12] Niesen-Vertommen SL, Taunton JE, Clement DB, Mosher RE (1992). The effect of eccentric versus concentric exercise in the management of Achilles tendonitis. Clin J Sport Med.

[B13] Neeter C, Thomeé R, Silbernagel KG, Thomeé P, Karlsson J (2003). Iontophoresis with or without dexamethazone in the treatment of acute Achilles tendon pain. Scand J Med Sci Sports.

[B14] Paoloni JA, Appleyard RC, Nelson J, Murrell GA (2004). Topical glyceryl trinitrate treatment of chronic noninsertional achilles tendinopathy. A randomized, double-blind, placebo-controlled trial. J Bone Joint Surg Am.

[B15] Lowdon A, Bader DL, Mowat AG (1984). The effect of heel pads on the treatment of Achilles tendinitis: a double blind trial. Am J Sports Med.

[B16] Peers K (2003). Extracorporeal Shock Wave Therapy in chronic Achilles and patellar tendinopathy.. Leuven University Press,.

[B17] Robinson JM, Cook JL, Purdam C, Visentini PJ, Ross J, Maffulli N, Taunton JE, Khan KM (2001). The VISA-A questionnaire: a valid and reliable index of the clinical severity of Achilles tendinopathy. Br J Sports Med.

[B18] Beaton DE, Bombardier C, Guillemin F, Ferraz MB (2000). Guidelines for the process of cross-cultural adaptation of self-report measures. Spine.

[B19] Grimby G (1986). Physical activity and muscle training in the elderly. Acta Med Scand Suppl.

[B20] Tallon C, Coleman BD, Khan KM, Maffulli N (2001). Outcome of surgery for chronic Achilles tendinopathy. A critical review. Am J Sports Med.

[B21] Paavola M, Orava S, Leppilahti J, Kannus P, Jarvinen M (2000). Chronic Achilles tendon overuse injury: complications after surgical treatment. An analysis of 432 consecutive patients. Am J Sports Med.

[B22] Alfredson H, Pietila T, Jonsson P, Lorentzon R (1998). Heavy-load eccentric calf muscle training for the treatment of chronic Achilles tendinosis. Am J Sports Med.

[B23] Koenig MJ, Torp-Pedersen S, Qvistgaard E, Terslev L, Bliddal H (2004). Preliminary results of colour Doppler-guided intratendinous glucocorticoid injection for Achilles tendonitis in five patients. Scand J Med Sci Sports.

[B24] Öhberg L, Alfredson H (2003). Sclerosing therapy in chronic Achilles tendon insertional pain-results of a pilot study. Knee Surg Sports Traumatol Arthrosc.

[B25] Öhberg L, Lorentzon R, Alfredson H (2001). Good clinical results but persisting side-to-side differences in calf muscle strength after surgical treatment of chronic Achilles tendinosis: a 5-year follow-up. Scand J Med Sci Sports.

