# Supplementary material for: Cross-cultural adaptation of the VISA-A questionnaire, an index of clinical severity for patients with Achilles tendinopathy, with reliability, validity and structure evaluations
Source: BMC Musculoskelet Disord. 2005 Mar 6;6:12. doi: 10.1186/1471-2474-6-12 (PMC555595; doi:10.1186/1471-2474-6-12)
Supplement: Additional File 1 — VISA-A-S questionnaire The Swedish version of the VISA-A questionnaire in MICROSOFT WORD format. [file 1471-2474-6-12-S1.doc]

# VISA-A-S FRÅGEFORMULÄR – utvärdering av hälsenebesvär

**Namn:__________________________ Datum:_______ Skadad hälsena: Höger / Vänster**

**I DETTA FRÅGEFORMULÄR SYFTAR ORDET *SMÄRTA***

**SPECIFIKT PÅ SMÄRTA I HÄLSENAN.**

1. När Du stiger upp på morgonen, under hur många minuter upplever du då stelhet i hälsenan?

Poäng

**100 min**  **0 min**

| **100 min** | **90 min** | **80 min** | **70 min** | **60 min** | **50 min** | **40 min** | **30 min** | **20 min** | **10 min** | **0 min** |
| --- | --- | --- | --- | --- | --- | --- | --- | --- | --- | --- |

0 1 2 3 4 5 6 7 8 9 10

2. När du väl är igång under dagen, har du då smärta när du stretchar hälsenan maximalt över en trappkant? (med sträckt knä)

**kraftig/ ingen**

|  |  |  |  |  |  |  |  |  |  |  |
| --- | --- | --- | --- | --- | --- | --- | --- | --- | --- | --- |

**svår smärta smärta**

0 1 2 3 4 5 6 7 8 9 10

3. Om du går på plant underlag i 30 minuter, får du då ont i hälsenan inom de närmaste 2 timmarna? (Om du på grund av smärta inte kan gå på plant underlag i 30 minuter, sätt 0 på denna fråga).

Poäng

**kraftig/ ingen**

|  |  |  |  |  |  |  |  |  |  |  |
| --- | --- | --- | --- | --- | --- | --- | --- | --- | --- | --- |

**svår smärta smärta**

0 1 2 3 4 5 6 7 8 9 10

4. Får du ont i hälsenan vid normal gång nedför en trappa?

**kraftig/ ingen**

|  |  |  |  |  |  |  |  |  |  |  |
| --- | --- | --- | --- | --- | --- | --- | --- | --- | --- | --- |

**svår smärta** **smärta**

0 1 2 3 4 5 6 7 8 9 10

5. Om Du gör 10 tåhävningar (på ett ben) på plant underlag, får du då ont i hälsenan under tiden eller direkt efter?

**kraftig/ ingen**

**svår smärta smärta**

|  |  |  |  |  |  |  |  |  |  |  |
| --- | --- | --- | --- | --- | --- | --- | --- | --- | --- | --- |

0 1 2 3 4 5 6 7 8 9 10

6. Hur många hopp på ett ben kan du göra utan att få ont i hälsenan?

Poäng

Poäng

Poäng

|  |  |  |  |  |  |  |  |  |  |  |
| --- | --- | --- | --- | --- | --- | --- | --- | --- | --- | --- |

Poäng

**0 10**

0 1 2 3 4 5 6 7 8 9 10

7. Utövar du för närvarande någon idrott eller annan fysisk aktivitet?

0 Inte alls

Poäng

4 Anpassad/begränsad träning och/eller anpassad/begränsad tävling

7 Tränar och/eller tävlar för fullt, men inte på samma nivå som innan hälsenebesvären började.

10 Tävlar på samma nivå eller högre nivå som innan hälsenebesvären började.

8. Besvara antingen A, B eller C i denna fråga.

- Om du inte har någon smärta under aktivitet som belastar hälsenan, besvara

endast **fråga A.**

- Om du har smärta under aktivitet som belastar hälsenan, men smärtan hindrar dig inte från att fullfölja aktiviteten, besvara endast **fråga B.**
- Om du har smärta som hindrar dig från att slutföra aktivitet som belastar hälsenan, besvara endast **fråga C.**

A. Om du inte har någon smärta under aktivitet som belastar hälsenan, hur länge kan du då delta i aktiviteten?

0 min 1-10 min 11-20 min 21-30 min >30 min

Poäng

0 7 14 21 30

ELLER

B. Om du har smärta under aktivitet som belastar hälsenan, men smärtan hindrar dig inte från att fullfölja aktiviteten, hur länge kan du då delta i aktiviteten?

Poäng

0 min 1-10 min 11-20 min 21-30 min >30 min

0 4 10 14 20

Eller

C. Om du har smärta som hindrar dig från att slutföra aktivitet som belastar hälsenan, hur länge kan du då delta i aktiviteten?

0 min 1-10 min 11-20 min 21-30 min >30 min

Poäng

0 2 5 7 10
